# A desmoid tumor with fluorodeoxyglucose accumulation arising from the anastomotic site of postoperative gastric cancer: a case report

**DOI:** 10.1186/s13256-022-03635-w

**Published:** 2022-11-16

**Authors:** Kento Kawashima, Kazuhiro Hiramatsu, Takehito Kato, Masahide Fukaya, Taro Aoba, Atsuki Arimoto, Takayuki Yamaguchi

**Affiliations:** Department of General Surgery, Toyohashi Municipal Hospital, 50 Hachikennishi, Aotake-cho, Toyohashi, Aichi 441-8570 Japan

**Keywords:** Desmoid tumors, FDG accumulation, Fibromatosis

## Abstract

**Background:**

Desmoid tumors are extremely rare borderline benign and malignant tumors that do not exhibit accumulation on fluorodeoxyglucose positron emission tomography–computed tomography. In the present study, we report a rare case of a desmoid tumor with fluorodeoxyglucose accumulation at the anastomotic postoperative gastric cancer site.

**Case presentation:**

A 68-year-old Japanese man underwent robot-assisted laparoscopic distal gastrectomy for early-stage gastric cancer in 2019. The pathological diagnosis was stage IA cancer, and no adjuvant chemotherapy was administered. Two years after surgery, a soft mass appeared on the greater curvature side of the anastomosis on computed tomography. Fluorodeoxyglucose positron emission tomography–computed tomography revealed fluorodeoxyglucose accumulation, which suggested a malignancy; therefore, surgery was performed for diagnostic treatment. The histopathological findings led to the diagnosis of a desmoid tumor. The patient has not experienced recurrence to date.

**Conclusions:**

In the present study, we encountered a desmoid tumor arising from the anastomotic site of a postoperative gastric cancer. This case is rare as fluorodeoxyglucose positron emission tomography–computed tomography showed fluorodeoxyglucose accumulation in the desmoid tumor, and a preoperative diagnosis could not be reached. We hope that further studies will improve the accuracy of preoperative diagnosis.

## Background

Desmoid tumors are extremely rare benign or malignant borderline tumors, and mechanical stimuli such as surgery are considered triggers for their development. Preoperative diagnosis with standard imaging modalities is difficult, and desmoid tumors do not exhibit any accumulation of fluorodeoxyglucose on positron emission tomography–computed tomography (FDG-PET/CT). In the present study, we report the case of a desmoid tumor with fluorodeoxyglucose (FDG) accumulation at the anastomotic site following robot-assisted laparoscopic distal gastrectomy, along with a review of the literature.

## Case presentation

A 68-year-old Japanese man was found to have an extensive type 0–IIc lesion in the upper gastric body extending up to the hypogastric lesser curvature on upper gastrointestinal endoscopy in September 2019. Biopsy revealed a poorly differentiated adenocarcinoma; the patient was referred to our department for surgery. He had hypertension and hyperlipidemia. However, he had no family history of malignancy. The patient underwent robot-assisted laparoscopic distal gastrectomy, D1+ lymph node dissection, and Roux-en-Y reconstruction in November 2019. The patient had a good postoperative course and was discharged 6 days postoperatively.

**Fig. 1:**
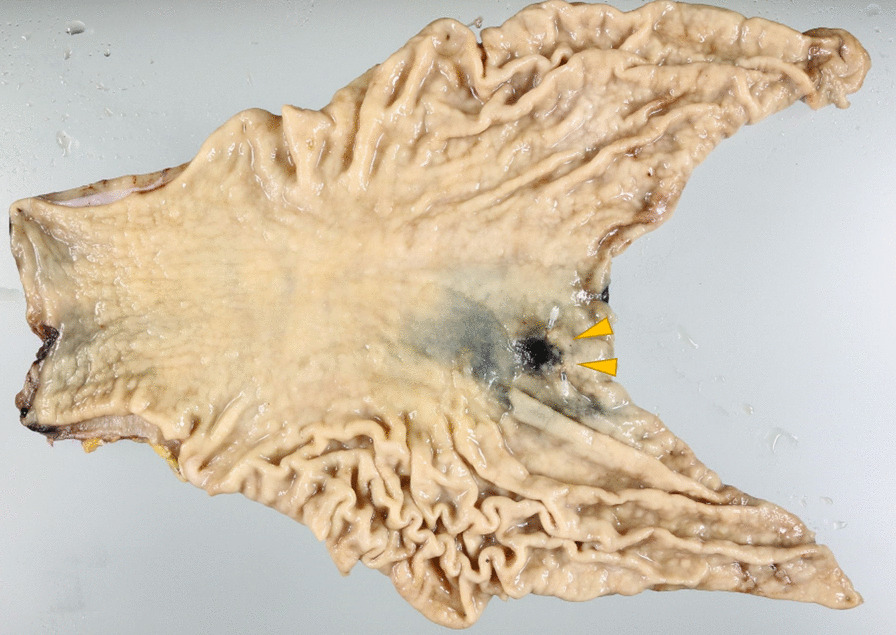
First surgical specimen. The arrows show the tumor. Lesion with shallow depression extending from the upper gastric body to the hypogastric lesser curvature

The initial surgical specimen findings were as follows (Fig. 1): a 50 × 35-mm 0–IIc lesion with shallow depression ranging from the upper gastric body to the hypogastric lesser curvature. Histopathological examination revealed a poorly differentiated adenocarcinoma with negative resection margins that were staged IA (pT1aN0M0) according to the Union for International Cancer Control (UICC) TNM Classification of Malignant Tumors, 8th edition.

**Fig. 2:**
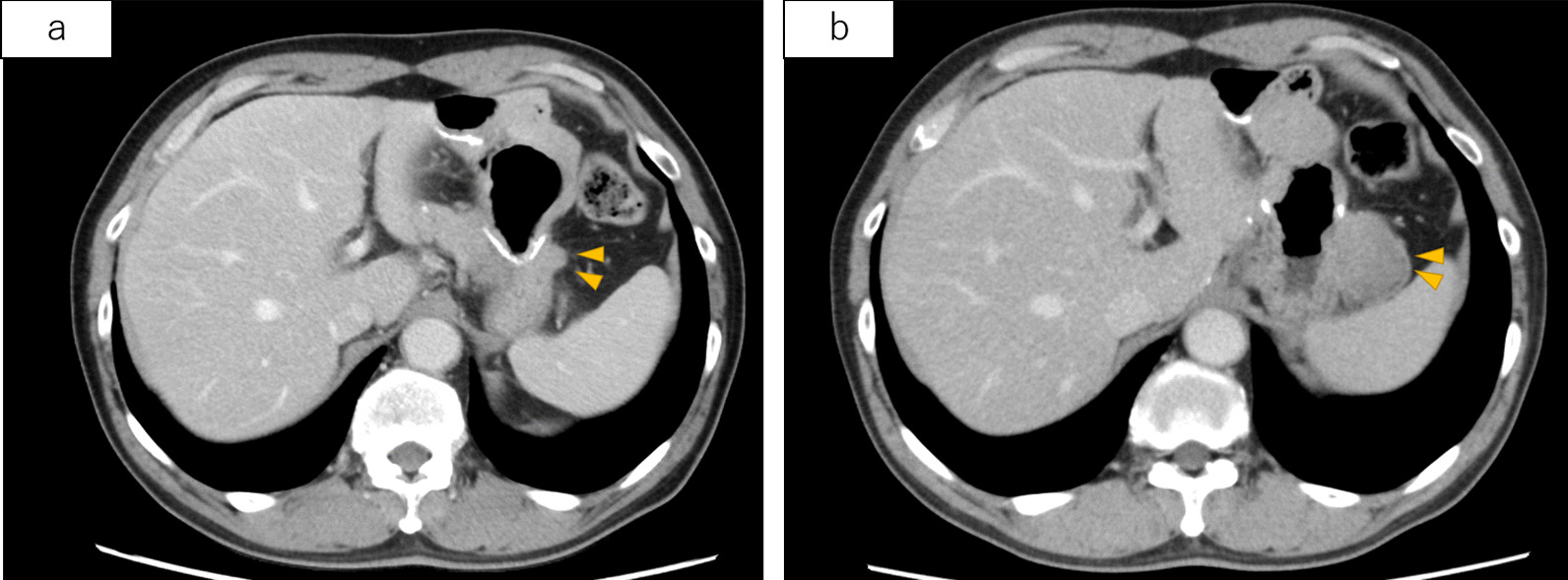
One year postoperatively (**a**), 2 years postoperatively (**b**). The soft tissue mass on the greater curvature side of the residual stomach is enlarged. The arrows show the soft tissue mass

The patient was followed-up without adjuvant chemotherapy; however, a 38 × 36 mm soft tissue mass was noted on the greater curvature side of the residual stomach during computed tomography (CT) 2 years after the procedure. Although partial thickening of the gastric wall was seen retrospectively in a 1-year postoperative CT scan, it was reviewed within normal limits by a radiologist at that time (Fig. 2). No evidence of recurrent metastasis was found at other sites.

The blood test findings were as follows: carcinoembryonic antigen (CEA), 1.8 ng/mL; carbohydrate antigen 19-9 (CA19-9), 8.5 U/mL; and soluble IL-2 receptor, 396 U/mL. Colonoscopy had been performed prior to the first surgery; hence, it was not performed again.

**Fig. 3:**
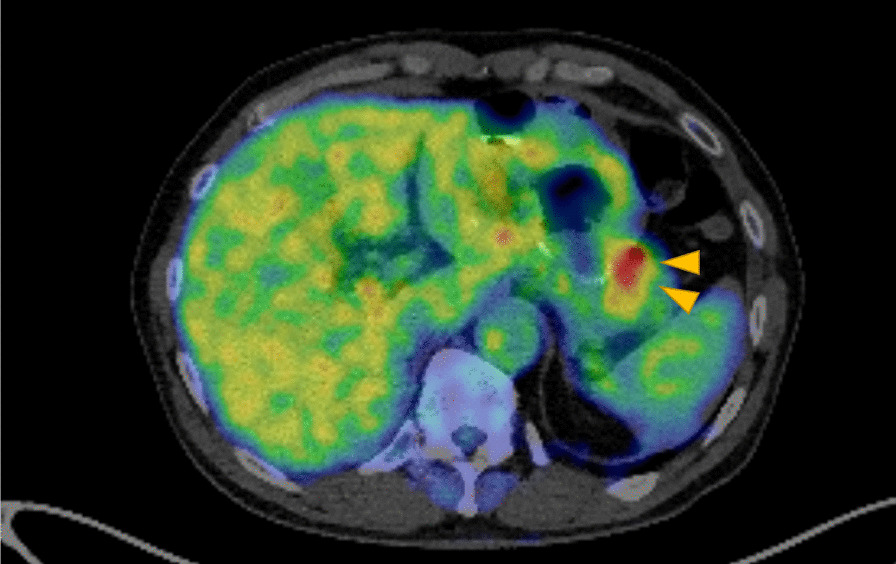
Fluorodeoxyglucose positron emission tomography–computed tomography. The soft tissue mass on the greater curvature side of the residual stomach shows FDG accumulation (SUVmax 5.25). The arrows show the soft tissue mass. *FDG* fluorodeoxyglucose

The FDG-PET/CT findings were as follows (Fig. 3): The soft tissue mass on the left side of the residual stomach showed moderate FDG accumulation (SUVmax, 5.25). No FDG accumulation was observed in other areas. Malignant lesions, including recurrent gastric cancer and regional lymph node metastasis, were suspected; however, qualitative diagnosis was difficult. No abnormal accumulation indicating recurrence and metastasis occurred at other sites.

**Fig. 4:**
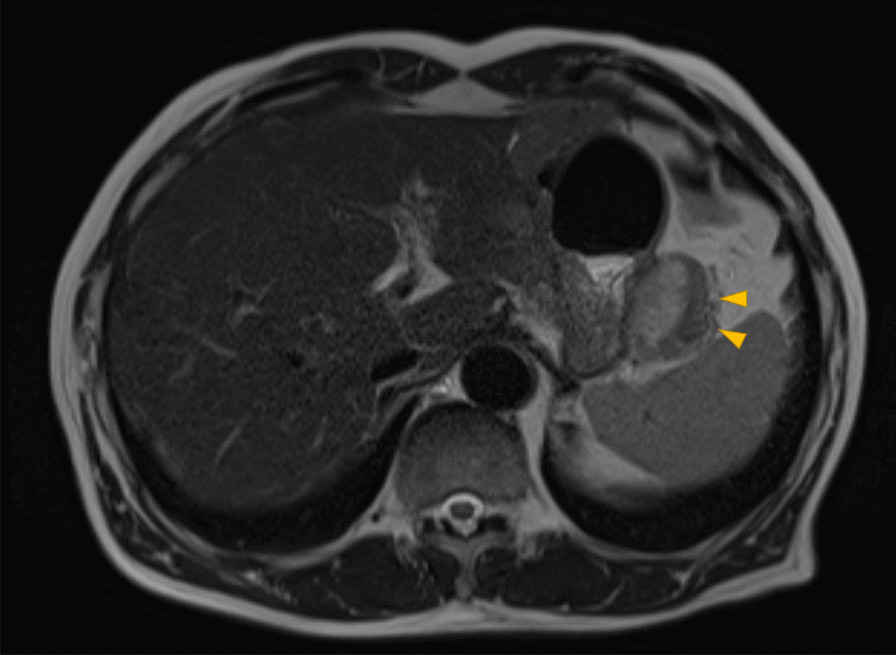
Magnetic resonance imaging. T2-weighted image showing a heterogeneous high signal. The arrows show the soft tissue mass

Contrast-enhanced magnetic resonance imaging (MRI) of the abdomen revealed the following findings (Fig. 4): the soft tissue mass on the greater curvature side of the residual stomach exhibited a high, heterogenous signal on T2-weighted images and a low signal on T1-weighted images, and contrast imaging revealed a slow contrast effect. Other malignant lesions such as gastric cancer recurrence, lymph node metastasis, and a gastrointestinal stromal tumor (GIST) were considered as possible differential diagnoses in addition to desmoid tumors; however, none of the findings were typical, and qualitative diagnosis was difficult.

**Fig. 5:**
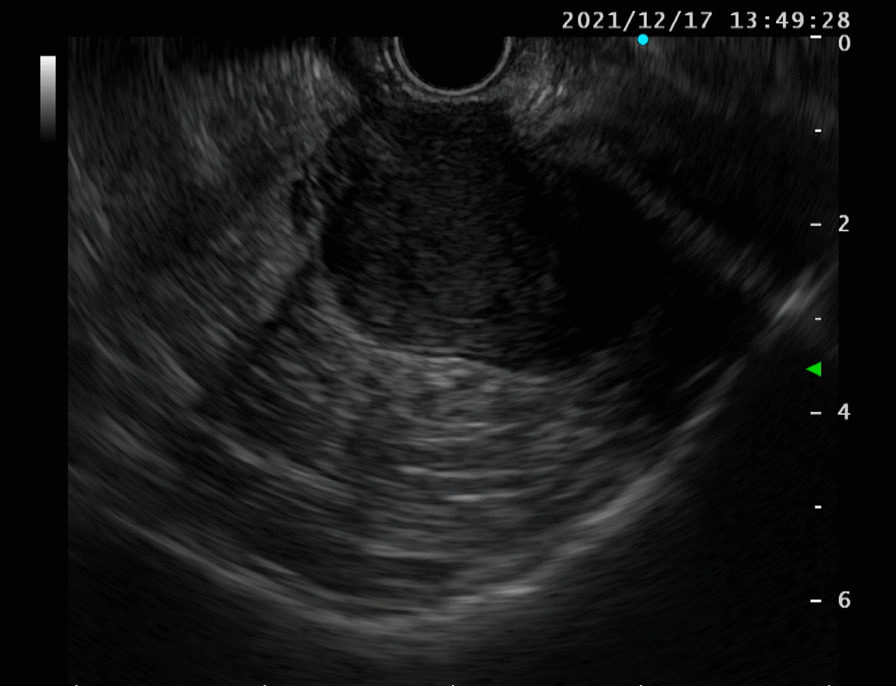
Endoscopic ultrasound-guided fine-needle aspiration. A borderline oligo-hypoechoic mass in the splenic hilum

Upper gastrointestinal endoscopic findings were as follows (Fig. 5): no mass lesions were present in the remnant stomach. Transgastric ultrasonography revealed a 43 × 30 mm borderline oligo-hypoechoic mass in the splenic hilum, which was biopsied using endoscopic ultrasound-guided fine needle aspiration (EUS-FNA).

The biopsy results were as follows: the tumor contained multiple spindle-shaped cells, and immunostaining for CD34, c-kit, desmin, S-100, and β-catenin was performed to distinguish spindle-shaped tumors; however, no significant positive findings were obtained. The biopsy specimen did not allow us to estimate the histological type of the tumor as it did not conclusively suggest a GIST, smooth myxoid, schwannoma, or desmoid tumor.

Based on the above-mentioned examination results, local recurrence of gastric cancer, lymph node metastasis, a GIST, a smooth myxoid, schwannoma, and a desmoid tumor were regarded as possible differential diagnoses. Although the possibility of a benign tumor remained, a definitive diagnosis could not be made histopathologically, and moderate FDG accumulation could not rule out a malignant lesion; thus, we decided to operate for diagnostic treatment. Preoperative CT revealed a suspicious finding of splenic arteriovenous invasion; therefore, total residual gastrectomy and splenectomy were planned.

The surgical findings were as follows: the abdomen was opened via a median upper abdominal incision. A 50 × 40 mm soft tissue mass originating from the gastrojejunal anastomosis involving the spleen and splenic arteriovenous was observed on the greater curvature side of the anastomosis. Additionally, a partial invasion of the pancreas was observed. The remaining stomach, spleen, splenic artery and vein, and part of the pancreas, including the gastrojejunal anastomosis, along with the soft tissue mass, were removed as a single lump. Regarding reconstruction, only esophago-jejunal anastomosis was performed, sparing previous jejuno-jejunal anastomosis.

**Fig. 6:**
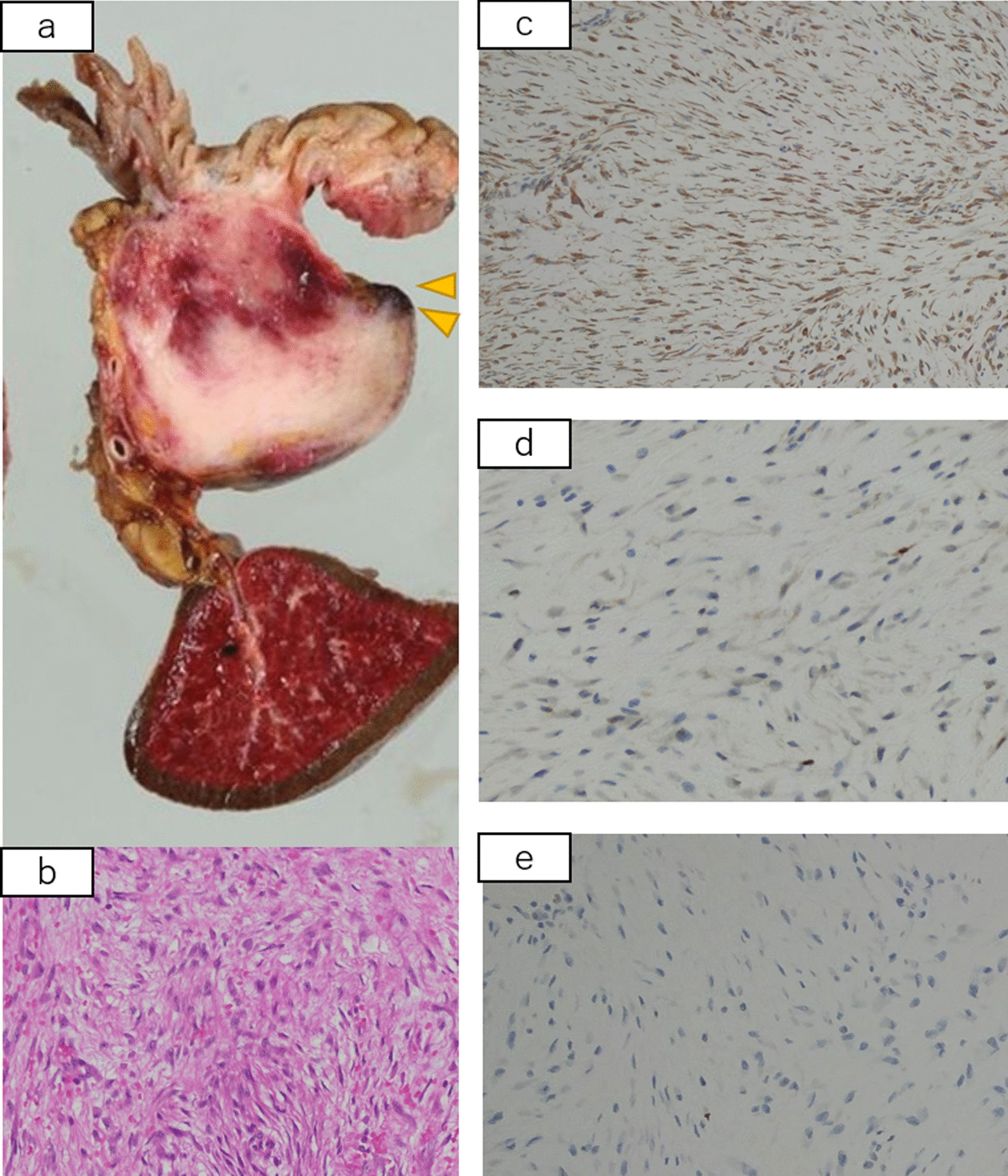
Pathological findings. The arrows show the tumor. Tumor growth involving anastomosis is observed (**a**). Hematoxylin–eosin staining showing spindle-shaped cell proliferation without atypia (**b**). Immunostaining is positive for vimentin (**c**) and β-catenin (**d**) expression and negative for Ki-67 (**e**) expression

The operation time was 279 minutes, and blood loss was 1100 mL; however, no blood transfusion was required. Pathological findings of the resected specimen were as follows (Fig. 6): a 45 × 40 × 40-mm soft tissue mass extending from the muscularis propria to the serosa at the gastrojejunal anastomosis. The tumor was a white, substantial nodule, and its growth involved the gastrojejunal anastomosis. Although fibroblasts and spindle-shaped cells were observed inside the tumor, the Ki-67 index was about 2%, and atypia was not observed. Immunostaining was positive for vimentin, desmin, and β-catenin and negative for α-SMA, S-100, CD34, c-kit, STAT6, estrogen receptor (ER), and progesterone receptor (PgR). Based on the above, a desmoid tumor was diagnosed.

## Postoperative course

The patient developed a postoperative pancreatic fistula that resolved after drainage. He was discharged from the hospital on postoperative day 16 and has not experienced any recurrence for 6 months.

## Discussion and conclusions

Desmoid tumors are a type of fibromatosis that are on the borderline between being benign and malignant, with an annual incidence of 2–4 per million people [1]. Pathologically, desmoid tumors are characterized by the proliferation of differentiated fibroblasts, presence of collagen fibers between cells, invasive growth patterns, absence of malignant features, and local recurrence without metastasis [2]. The three types of desmoid lesions are as follows: extra-abdominal, abdominal wall, and intra-abdominal desmoid, and the frequency of their occurrence is 43%, 49%, and 8%, respectively [1]. The etiology of desmoid tumors is unknown; however, genetic familial adenomatous polyposis (FAP), trauma, history of abdominal surgery, and female hormones may be involved [3].

MRI has excellent tissue resolution and is the most useful imaging technique for diagnosing desmoid tumors, with T1-weighted images showing equal signals and T2-weighted images showing high or heterogeneous signals [4]. However, desmoid tumors have no characteristic imaging findings, and making a definitive preoperative diagnosis is considered extremely difficult. FDG-PET/CT has recently been performed in some cases. As a desmoid tumor is a benign tumor, FDG accumulation is generally low. Suzumura *et al.* have reported 12 cases of intra-abdominal desmoid tumors with FDG uptake on PET/CT. They have reported that only one case showed high accumulation; however, the remaining 11 cases showed only mild accumulation with the maximum standardized uptake value (SUVmax) ranging between 1.95 and 3.9 [5]. The cause of hyperaccumulation of FDG is not clear; however, some reports indicate that SUVmax increased with tumor growth, whereas other reports suggest that SUVmax reflects the efficacy of drug therapy [6, 7]. Therefore, hyperaccumulation of FDG in the present case may suggest that the desmoid tumor tended to increase in size.

As our patient was a postoperative gastric cancer patient, the moderate accumulation on FDG-PET/CT could not be ruled out as a recurrence of gastric cancer or lymph node metastasis. We decided to perform EUS-FNA in this case because some studies have mentioned that preoperative EUS-FNA should have been performed and some have reported that the diagnosis was made using needle biopsy [8, 9]. In Japan, EUS-FNA is not recommended in cystic masses because it can cause peritoneal dissemination; however, in this case, we performed EUS-FNA because of the suspicion of a substantial mass [10]. Although we could only identify spindle-shaped tumors, we could not reach a definitive diagnosis. If the gastric cancer stage had been higher, chemotherapy may have been considered an option due to the possibility of recurrence of gastric cancer. However, in the present case, the stage was so early that recurrence was unlikely and surgery was the only treatment option for diagnostic purposes.

The local recurrence rate of desmoid tumors is extremely high, ranging between 56% and 86% [11]. Therefore, the possibility of recurrence should be considered, and the patient should be carefully monitored.

In the present study, we encountered a desmoid tumor arising from the anastomotic site of a postoperative gastric cancer. The present case is rare in that FDG-PET/CT showed desmoid tumor accumulation, and a preoperative diagnosis could not be made. We hope that further studies will improve the accuracy of preoperative diagnosis.

## Data Availability

The datasets used and/or analyzed during the current study are available from the corresponding author on reasonable request.

## Abbreviations

FDG-PET/CT: Fluorodeoxyglucose positron emission tomography–computed tomography
CEA: Carcinoembryonic antigen
CA19-9: Carbohydrate antigen 19-9
MRI: Magnetic resonance imaging
GIST: Gastrointestinal stromal tumor
EUS-FNA: Endoscopic ultrasound-guided fine-needle aspiration
ER: Estrogen receptor
PgR: Progesterone receptor
FAP: Familial adenomatous polyposis
SUVmax: Maximum standardized uptake value
